# Running Speed Can Be Predicted from Foot Contact Time during Outdoor over Ground Running

**DOI:** 10.1371/journal.pone.0163023

**Published:** 2016-09-20

**Authors:** Cornelis J. de Ruiter, Ben van Oeveren, Agnieta Francke, Patrick Zijlstra, Jaap H. van Dieen

**Affiliations:** Faculty of Behavioural and Movement Sciences, Research Institute MOVE, Vrije Universiteit Amsterdam, Amsterdam, The Netherlands; West Virginia University School of Medicine, UNITED STATES

## Abstract

The number of validation studies of commercially available foot pods that provide estimates of running speed is limited and these studies have been conducted under laboratory conditions. Moreover, internal data handling and algorithms used to derive speed from these pods are proprietary and thereby unclear. The present study investigates the use of foot contact time (CT) for running speed estimations, which potentially can be used in addition to the global positioning system (GPS) in situations where GPS performance is limited. CT was measured with tri axial inertial sensors attached to the feet of 14 runners, during natural over ground outdoor running, under optimized conditions for GPS. The individual relationships between running speed and CT were established during short runs at different speeds on two days. These relations were subsequently used to predict instantaneous speed during a straight line 4 km run with a single turning point halfway. Stopwatch derived speed, measured for each of 32 consecutive 125m intervals during the 4 km runs, was used as reference. Individual speed-CT relations were strong (r^2^ >0.96 for all trials) and consistent between days. During the 4km runs, median error (ranges) in predicted speed from CT 2.5% (5.2) was higher (P<0.05) than for GPS 1.6% (0.8). However, around the turning point and during the first and last 125m interval, error for GPS-speed increased to 5.0% (4.5) and became greater (P<0.05) than the error predicted from CT: 2.7% (4.4). Small speed fluctuations during 4km runs were adequately monitored with both methods: CT and GPS respectively explained 85% and 73% of the total speed variance during 4km runs. In conclusion, running speed estimates bases on speed-CT relations, have acceptable accuracy and could serve to backup or substitute for GPS during tarmac running on flat terrain whenever GPS performance is limited.

## Introduction

Speed is the primary performance parameter for running. In practice, training schedules are largely based on running speed, often in combination with heart rate. Running speed is not only of interest for professional and amateur sportsmen, but also for millions of recreational runners all over the world. The global position system (GPS) is often used for monitoring outdoor running speed and GPS indeed provides quite accurate speed estimates, especially for running in straight lines. However, speed accuracy declines when sharp turns or bends are made [1], or rapid changes in speed occur [2]. Moreover, GPS signals are sometimes poorly received, for instance when buildings block the signals and obviously GPS cannot be used indoors. As an alternative, small inertial sensors can be used to provide runners with estimates of their running speed. Such speed estimations can also serve as backup whenever GPS reception is poor or absent (indoors). Some of the commercially available devices have been validated and seem to provide reasonably accurate measures of running speed, at least under laboratory conditions [3, 4]. However, the number of published validation studies is limited. Importantly, the protocols and algorithms used to derive running speed from the inertial sensors in these commercial devices are proprietary, consequently it is unclear how running speed is estimated. Direct integration of these sensors’ acceleration signals to obtain measures of running speed is not yet accurate enough [5].

It has been shown that stride frequency (SF) increases with speed in a near linear way [6–8] and acceptable relations between SF obtained with inertial sensors worn on the chest and speed have been found while running 50m stretches [9]. However, in the laboratory, much stronger relations have been found between foot contact time (CT) and speed [10, 11] even across species [12]. Therefore CT in particular seems a promising variable from which speed estimates can be derived. However, outdoor conditions (wind etc.) could decrease the strength of the relations between speed and CT compared to those obtained in the laboratory. Moreover, data on the reproducibility of the relations between speed and CT, even under laboratory conditions, are lacking. Therefore, the first goal of the present study was to establish these relationships at different days during short (125m) stretches, run outdoors on tarmac at different (but constant) speeds.

Runners are often interested to receive accurate feedback on small changes in speed and many strive to maintain a constant speed during endurance runs. Therefore, the second goal of this study was to investigate how accurate speed and small changes thereof can be predicted from previous established relations between speed and CT. To this end, participants ran 4 km on two days, during which average speed for every 125m interval was calculated from stopwatch timing and used as a reference for both CT and GPS derived speed. Both methods were compared with special attention for the start, finish and stretches around the running point, where GPS performance is expected to decrease.

## Materials and Methods

### Participants

Fourteen healthy subjects (8 men, 6 women) participated in this study (Table 1). We included participants who were recreationally active (2–15 hours per week) in various sports. Most were not specifically endurance trained and two did not perform any run training at all (no. 4, 9). Two participants (no. 2 and 5) were well trained and ran about 50 km weekly. This contributed to considerable differences in running performance (4 km speed) among participants (Table 2). Prior to participation, experimental procedures, risks and aims of the study were explained and all subjects provided written informed consent. The study was conducted according to the principles of the Declaration of Helsinki and was approved (approval number: 2014–35, April 15th) by the ethics committee of the Department of Human Movement Sciences, Vrije Universiteit, Amsterdam, the Netherlands. Participants were recruited during the last two weeks of April 2014.

**Table 1:** Participant characteristics and weather conditions.

	age	height	body	BMI	leg	sex	day1		day2	
			mass		length		wind	T	wind	T
	(year)	(m)	(kg)	(kg^.^m^-2^)	(m)		(m^.^s^-1^)	(°C)	(m^.^s^-1^)	(°C)
1	24	1.74	65	21.5	1.18	f	w-10	9	se-4	10
2	28	1.80	67	20.7	1.24	m	sw-8	12	ne-6	14
3	22	1.66	59	21.4	1.14	f	ne-6	15	sw-2	14
4	23	1.91	85	23.3	1.32	m	sw-10	13	w-7	12
5	26	2.00	73	18.3	1.32	m	sw-9	17	n-4	13
6	51	1.84	85	25.1	1.28	m	sw-8	15	n-6	13
7	24	1.80	77	23.8	1.24	f	n-6	8	sw-9	14
8	23	1.91	78	21.4	1.33	m	se-4	10	s-2	17
9	23	1.80	70	21.6	1.28	f	e-5	23	s-9	19
10	26	1.83	70	20.9	1.27	m	w-3	15	sw-8	15
11	24	1.89	85	23.8	1.32	m	w-10	9	n-6	8
12	25	1.68	56	19.8	1.15	f	w-6	11	s-8	25
13	17	1.69	63	22.1	1.18	f	sw-1	11	sw-5	15
14	22	1.86	70	20.2	1.27	m	s-5	17	n-5	13
mean	25	1.82	71	21.7	1.25		6.5	13.2	5.6	14.4
s	7	.09	9.1	1.7	0.07		2.8	4.1	2.5	4.1

Age, height, body mass, Body Mass Index (BMI), leg length (including the foot), sex (male female), wind direction (east, west, south, north), followed by wind speed and temperature on both days.

**Table 2:** Error of different methods for running speed determination during two 4 km runs.

	speed		error (%) compared to			
	(m^.^s^-1^)		stopwatch-speed			
			CT		GPS	
run no.	1	2	1	2	1	2
participant						
1	3.2	3.3	1.9	1.4	1.2	1.9
2	4.5	4.6	3.9	2.9	1.6	2.3
3	2.7	2.5	1.0	0.8	1.5	1.8
4	3.3	2.8	2.6	4.8	2.2	1.8
5	4.8	4.7	5.4	6.7	2.2	1.4
6	3.5	3.7	2.2	2.4	1.9	1.4
7	2.5	2.1	1.4	1.5	1.6	1.4
8	4.0	4.1	1.6	1.7	1.7	1.3
9	2.6	2.5	3.3	1.3	2.0	1.4
10	4.2	4.1	3.1	1.6	1.9	1.8
11	3.4	3.4	4.4	2.8	1.3	1.1
12	3.1	3.0	4.2	1.2	1.2	1.6
13	3.5	3.3	3.7	3.1	1.6	1.3
14	3.3	3.8	1.6	3.3	1.0	2.6
median	3.4	3.4	2.9	2.1	1.6	1.5
range	2.3	2.6	4.4	5.9	1.2	1.5
median comb.	3.4		2.5		1.6*	
range comb.	2.5		5.2		0.8	

Running speed (two runs), predicted from contact time (CT) and GPS are compared to speed determined with stopwatch timing as reference. Individual mean 4 km speed for both runs is shown in columns 2 and 3. Individual root of squared differences averaged over the thirty-two 125m intervals of each 4 km run are shown in the left four columns. In the bottom rows the median and ranges of the combined (comb.) data are depicted. The combined data for each participant are the mean values over both days.

* denotes different (P<0.05) from the other speed measure.

### Data collection

To assess consistency of speed predictions, each participant was tested on two separate days within a fourteen day period, but with at least 72 hours in between measurements. The local weather conditions (Table 1) during each run were retrieved from the website of the main Dutch meteorological institute (www.knmi.nl/index_en.html). Participants refrained from vigorous physical activity and avoided alcohol and caffeine in the 24 hours prior to test days, and wore the same sport shoes on both occasions. We aimed to obtain the best possible estimates for speed from the GPS device. To this end, runs were made on a flat and straight 2 km tarmac lane along the border of the Amsterdam 2014 World Championships rowing venue (East to West orientation). Paint marks were applied on the tarmac every 125m. During all runs, two researchers accompanied the runner on bike and one of them timed every 125m ‘lap’ with a stopwatch, from which average speed per 125m interval was calculated (stopwatch-speed).

The experimental protocol on both days was the same. Participants wore a Garmin Forerunner 620 sports watch with GPS function and a heart rate belt on the chest. In addition, two customized small wireless inertial sensors (MPU-9150, MEMS Motion Tracking^TM^ Device, InvenSense, San Jose, USA) with tri-axial accelerometers and gyroscopes, were firmly attached to the shoelaces with small rubber bands on the instep of both shoes. Data (500Hz sampling rate) were stored on an internal SD-card for off-line analysis to determine CTs of the feet. Participants started with a 10 min warm-up consisting a short 5 min run at a self-chosen pace and some stretching exercises. Thereafter, the first set of measurements (calibration runs) took place. These calibration runs consisted of six consecutive (no stops) 125m intervals run at different speeds in one direction, followed by 1 minute rest and the same six 125m intervals run in the opposite direction. The subjects were instructed to run each 125m interval at constant speed, representing respectively 40, 50, 20, 80, 50 and again 40% of their self-estimated maximum running intensity. This was done to guarantee that a sufficiently broad speed range would be covered for construction of the individual running speed-ground contact relations. They had to change to the next speed at each 125m mark in a few strides. After these calibration runs, there was 5 min rest before the participants ran 4 km from stand still with a single 180 degree turning point at 2 km and ending exactly in standstill at 4km. They completed the 4km at a self-selected speed but were not allowed to walk at any time.

### Data processing

The foot strike and toe off events, which give distinct peaks in the signals of the MEMS devices [6, 11], of each foot were automatically detected for every stride with customized software written in Matlab (R2011a, Mathworks, Natick, USA). CT was defined as the time interval between initial ground contact and toe off. To correct for sporadic outliers, the CT time series of both feet were filtered with a third order median filter. The mean values of both feet are presented.

For the calibration runs, stopwatch timing was used to calculate mean running speed for every 125 m- interval. This procedure resulted in CT values at twelve speeds for each participant. For each day, these twelve data points were fitted (least squares) with a power (y = cx^d^) function. The individual relations were subsequently used to predict instantaneous running speed from CT recorded for every stride during the 4 km runs. (CT-speed).

GPS position data (1 Hz) was differentiated to obtain instantaneous running speed based on GPS (GPS-speed). Subsequently, mean speed values for CT-speed and GPS-speed were calculated for the duration of all (thirty-two) 125m time intervals. In this manner, we could compare 125m-interval-speed estimates based on CT and GPS to the reference speed obtained with stopwatch timing for every 125m during the 4km runs.

For each of the thirty-two 125m-intervals during the 4km run, the root of squared differences of CT-speed and GPS-speed compared to the reference (stopwatch-speed) was calculated and expressed as a percentage of stopwatch-speed. These thirty-two values were averaged to obtain one error value for both methods of speed determination for each participant. Of these the group means were subsequently determined.

In addition to the potential error it is also highly relevant for a runner to know how well (small) changes in speed during an endurance run are predicted. To this end, we used two (CT and GPS) separate linear regressions for each participant, between the thirty-two speed values and the stopwatch speed of these thirty-two 125 m intervals. The variance explained (r^2^) by both speed estimates was calculated for each runner. Of these individual r^2^-values the group means were subsequently determined.

### Statistical Analysis

Data are presented as individual results, as means and standard deviation (s) or as median values with (range). The statistical analysis was done using the Statistical Package for Social Sciences (SPSS version 21.0; IBM, Armonk, New York, USA). The between days coefficient of variation (CV) was defined as (s^.^mean^-1^)^.^100%. Since the variance of the predicted speed was unequal between the methods, these data were first log-transformed. Student’s t-test was used (P<0.05 was significant) for pairwise comparisons. The anti-log values of the latter and their 95% confidence intervals (CI) were calculated, which provides the ratios of the average errors between both methods of speed estimation. Effect sizes (partial η^2^) are also reported.

## Results

### Calibration runs

**Fig 1:**
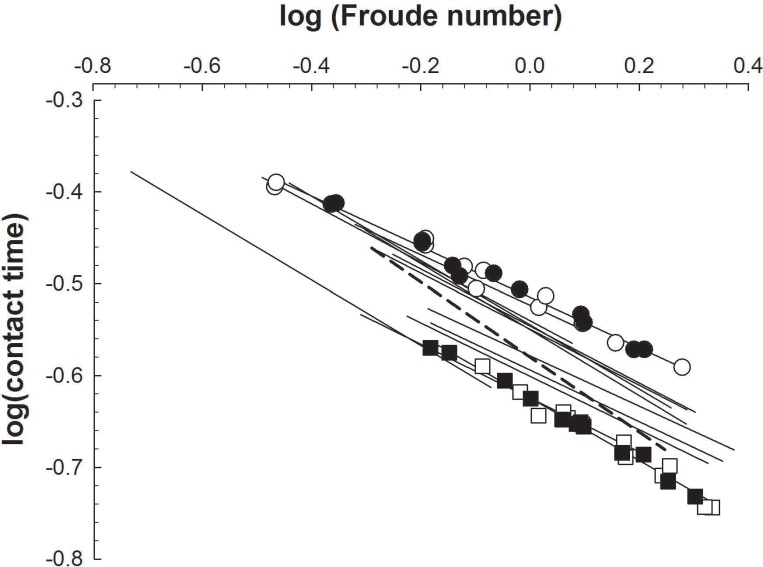
Ground contact time decreases with running speed. Logarithm of contact time as a function of the log-transformed Froude numbers (speed^2.^ 9.81^−1.^ leg length^-1^) of all 14 subjects. For all subjects the linear trendlines, for the data points of both days combined, are shown (all with r^2^>0.95). For clarity the actual data points (2x12, open and closed symbols for day 1 and 2 respectively) are only depicted for participant 2 (squares: y = -0.34x-0.62, r^2^ = 0.98) and 11 (circles: y = -0.28x-0.51, r^2^ = 0.98). In addition the regression line of participant no.3 has been dashed.

There were strong relations (y = c^.^CT^d^) between running speed and CT for all participants on both days, with r^2^ > 0.96 for all twenty-eight calibration runs and a mean of 0.98 ± 0.01 on both days. The values of coefficient c on the two days respectively were 0.59 ± 0.08 and 0.59 ± 0.08, while for coefficient d the respective values were -0.63 ± 0.08 and -0.63 ± 0.09. The coefficients of variation for the c and d coefficients respectively were 4.0 ± 3.6% and 5.3 ± 5.0%. Noteworthy is, that the individual relationships between CT and running speed were very similar between days, in spite of the differences in weather conditions between days (Table 1). The consistency of the individual relations is also depicted in Fig 1, where for each participant the data of both days are combined into a single relation (r^2^>0.95 for all participants). In Fig 1 log-transformed contact times for the runs are plotted as a function of the log-transformed Froude numbers (speed^2.^ 9.81^−1.^ leg length^-1^), to normalize speed for differences in leg length. These results show that even following this normalization there remained clear differences between participants.

### 4 km runs

Mean running speed ranged from 2.1 m^.^s^-1^ to 4.8 m^.^s^-1^ among participants (Table 2). There were no significant differences between days for any of the predicted speeds (P>0.05). Therefore the mean values of both days for each participant were analyzed. Median errors in predicted 125m-interval-speed for GPS and CT respectively were 1.6%, and 2.5% (Table 2). Median error in CT-speed was 1.5 times the error in GPS-speed (P<0.05, η^2^ = 0.44, CI: 1.1–2.0).

**Fig 2:**
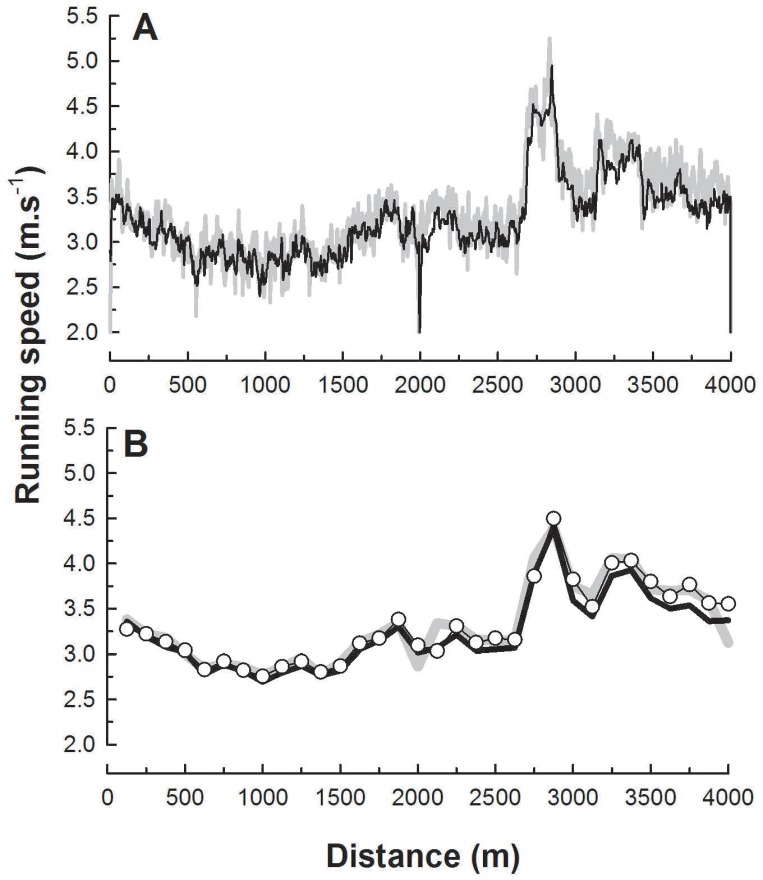
Fluctuations in running speed can be predicted from ground contact time. Running speed (participant 4, run 1) predicted with GPS (gray) and contact time (CT, black) as a function of the distance ran, resampled at 1Hz (A) and following averaging over intervals of 125m (bottom) for GPS (thick gray line), CT (black line) and stopwatch timing (open circles).

In the majority of runs, GPS-speed during the first, last and the middle two 125m-intervals (around the turning point at 2 km), deviated more from stopwatch-speed than CT-speed (see Fig 2 for example). When only these four intervals were included in the statistical analysis, median error for GPS-speed increased 3.0 times, from 1.6% with all intervals included (Table 2) to 5.0% with a range of 4.5% (P<0.05, η^2^ = 0.97, CI: 2.7–3.4). For CT, median error in predicted speed with only the first, last and the middle two 125m-intervals included was 2.7% with a range of 4.4%, which was 0.5 times that of the GPS prediction (P<0.05, η^2^ = 0.62, CI: 1.4–2.8).

Fig 2 (A) shows that fluctuations in running speed obtained from CT and GPS during 4 km running (resampled at 1 Hz for this figure) were very similar. This is also evident when speed for both methods was averaged for every 125m-interval (Fig 2(B)). Linear regression between these values and stopwatch-derived reference speed showed that CT (median r^2^ = 0.85, range 0.43) and GPS (median r^2^ = 0.73, range 0.56) respectively explained 85% and 73% of the individual running speed fluctuations during the 4 km runs.

## Discussion

### Calibration runs

The calibration runs indicated that CT was strongly related with speed. Comparable to the results for other species [12], a power function (y = c^.^CT^d^) provided an excellent fit for our speed-CT data (r^2^ = 0.98). The average c- (0.59) and d- coefficients (-0.63) are in good agreement with the respective values of 0.64 and -0.65 reported for men running on a treadmill which also had a similar coefficient of determination: r^2^ = 0.99 [12]. Thus, the present study clearly demonstrates that the previously reported relations in the laboratory hold during free running.

Our data (Fig 1) indicate that even following normalization of speed to leg length, there remained consistent differences in the contact time-running speed relations among our runners. One obvious factor contributing to such differences would be differences in foot strike patterns. Heel striking is accompanied by longer CTs than a mid-foot strike running technique e.g. [13]. We indeed observed that participant no. 11, running with long CTs, was a heel striker displaying a double burst of peaks in the sensor signals during landing. In contrast, participant no. 2, with short CTs (Fig 1), was a mid/front- foot striker who consistently displayed a single burst of peaks during landing. Moreover, participant no. 3 (dashed line in Fig 1), had double activity bursts in her signals at slower speeds and single bursts at faster speeds. This could indicate a change from heel to mid-foot landing with an increase in speed, as the percentage of mid foot strikers increases as running speed increases [14].

That we did find these differences between runners in our rather heterogeneous group of participants is not surprising, since even within a relatively homogeneous group of elite runners different relations between contact time and running speed on a treadmill were found for middle and long distance runners [6]. The relatively short CTs of our two most experienced runners (participants 2 and 5) may be coupled to a relative good running economy as found by others [8, 15, 16], but this is speculative.

### 4 km runs

The present results indicate that running speed estimates based on personalized speed-CT relations have acceptable accuracy during natural outdoor over ground running on flat terrain. On average GPS speed was more accurate than CT derived speed. However, in the beginning, end and around the turning point CT predictions were more accurate. Moreover, we optimized conditions for GPS performance in the present study. Therefore, it seems reasonable to conclude that CTs measured with a foot pod can serve as a reliable back up or substitute in conditions were GPS performance is less optimal (high buildings and/or many turns and bends) or even impossible (indoors).

Since the present conditions, open terrain straight course, were optimized for GPS performance, it is not surprising that errors in GPS-speed were low. Both GPS-speed and CT-speed had acceptable errors. Importantly, GPS-speed was less accurate than the CT-based speed prediction for the first, middle two (at the 2-km turning point) and end intervals. This is in line with the literature, where accuracy of GPS has been shown to decrease during acceleration and deceleration [1] and/or directional changes [2].

Stopwatch derived speed was used as a reference. The use of a cyclist to time the runner at each 125m interval probably introduced some error. Mean running speed was 3.4 m^.^s^-1^, which results in an average time of 36.7 s per 125m. Consequently in the most negative realistic scenario, when stopwatch would be pressed 0.5 s too early at the beginning as well as 0.5 s too late at the end of a 125m interval, this would result in a maximal error of 2.7% (0.09 m^.^s^-1^) in stopwatch-speed. Mean error in stopwatch-speed probably was lower than this. Moreover, GPS-speed and CT-speed still can be compared, since stopwatch-speed served as reference for both.

### Practical implications

The number of validation studies of commercially available foot pods that provide estimates of running speed is limited and these studies have been conducted under laboratory conditions. Moreover, internal data handling and algorithms used to derive speed are proprietary and thereby unclear [3, 4]. The present study shows that speed can be predicted from CT with acceptable accuracy. Even the relatively small speed fluctuations during runs of longer duration can be accurately predicted from CT. Because the relationships between CT and speed differ among participants, one careful individual calibration is necessary before speed can be predicted from CT. Since the speed-CT relations are virtually linear, for practical reasons two short runs (e.g. 200m) at different speeds would suffice. The present results show very good reproducibility of these relations under very different weather conditions. However, it remains to be established to what extent these relations change over time, necessitating recalibration. Our unpublished observations in some recreational runners indicate that over the course of one year, the relations remained stable and recalibration was not necessary. We currently do not know to what extent running surface and/or running on undulating terrain affect the speed-CT relations. Therefore, the present findings do not necessarily extend to runs on other surfaces and hilly terrain.

We found an increase in the error of GPS-speed around the turning point. Thus, when there are many turns and bends included in a course and/or when runs are performed in between high buildings, CT derived speed may even be more accurate than GPS derived speed and it certainly can serve as reliable backup whenever GPS receive is poor or absent (indoors).

## Conclusions

Similar to laboratory conditions, there are strong individual relations between running speed and CT during outdoor over ground running on flat terrain. The CT-speed relations were highly reproducible within individuals despite varying weather conditions. In addition, during 4km runs, speed estimates based on CT and GPS were found to be comparable. These results indicate that devices that estimate speed from CT measured with inertial sensors on the feet, can predict (changes in) running speed quite accurately and may be used in addition to GPS, when GPS function is compromised or not possible.

## Supporting Information

S1 File Individual data. (XLSX)
